# Towards a Kinetic Modeling of the Changes in the Electrical Properties of Cable Insulation during Radio-Thermal Ageing in Nuclear Power Plants. Application to Silane-Crosslinked Polyethylene

**DOI:** 10.3390/polym13244427

**Published:** 2021-12-16

**Authors:** Sarah Hettal, Simone Vincenzo Suraci, Sébastien Roland, Davide Fabiani, Xavier Colin

**Affiliations:** 1 Laboratoire Procédés et Ingénierie en Mécanique et Matériaux, Arts et Métiers Institute of Technology, CNRS, CNAM, HESAM University, 151 Boulevard de l’Hôpital, 75013 Paris, France; sarah.hettal@ensam.eu (S.H.); sebastien.roland@ensam.eu (S.R.); 2 LIMES, Department of Electrical, Electronic and Information Engineering, University of Bologna, Viale del Risorgimento, 40136 Bologna, Italy; simone.suraci@unibo.it (S.V.S.); davide.fabiani@unibo.it (D.F.)

**Keywords:** silane-crosslinked polyethylene, radio-thermal oxidation, analytical kinetic model, density, electrical properties, structural end-of-life criterion, lifetime prediction

## Abstract

The radio-thermal ageing of silane-crosslinked polyethylene (Si-XLPE) was studied in air under different γ dose rates (6.0, 8.5, 77.8, and 400 Gy·h^−1^) at different temperatures (21, 47, and 86 °C). The changes in the physico-chemical and electrical properties of Si-XLPE throughout its exposure were determined using Fourier transform infrared spectroscopy coupled with chemical gas derivatization, hydrostatic weighing, differential scanning calorimetry, dielectric spectroscopy and current measurements under an applied electric field. From a careful analysis of the oxidation products, it was confirmed that ketones are the main oxidation products in Si-XLPE. The analytical kinetic model for radio-thermal oxidation was thus completed with relatively simple structure–property relationships in order to additionally predict the increase in density induced by oxidation, and the adverse changes in two electrical properties of Si-XLPE: the dielectric constant $\varepsilon^{\prime }$ and volume resistivity R. After having shown the reliability of these new kinetic developments, the lifetime of Si-XLPE was determined using a dielectric end-of-life criterion deduced from a literature compilation on the changes in R with $\varepsilon^{\prime }$ for common polymers. The corresponding lifetime was found to be at least two times longer than the lifetime previously determined with the conventional end-of-life criterion, i.e., the mechanical type, thus confirming the previous literature studies that had shown that fracture properties degrade faster than electrical properties.

## 1. Introduction

Most nuclear power plants (NPPs) built in Europe in the 1980s and 1990s are now reaching their originally planned end-of-life, and electric utility companies are wondering whether it is possible to extend their operating life by a few more decades. As low-voltage electric cables are among the most critical components in NPPs, the monitoring of the health of their polymer insulation throughout the NPP’s operating life rapidly emerged as one major issue. For context, it should be mentioned that about 1500 km of low-voltage cable are used inside each NPP [1] to perform various functions, including power transmission, the control of equipment and instrumentation, and the communication of signal and data.

To reach this objective, two complementary monitoring approaches, based either on numerical simulations or on non-destructive testing in real time (i.e., on site), are being considered. The relevance of both approaches is tested within the framework of the H2020 TeamCables project, which is mainly focused on silane-crosslinked low-density polyethylene (Si-XLPE) insulation [2]. It should be mentioned that the silane crosslinking of polyethylene has recently become very popular in cable and pipe industries because it offers several advantages, including the ease of execution and low cost [3,4,5]. Indeed, this reaction does not require the use of additional specific equipment. Choosing the vinyl alkoxysilane molecule to be grafted along the polymer chain also offers the possibility of varying the chemical structure of the crosslinking junctions, and thus the possibility of adjusting the final properties of the polymer piece [3].

Since the early 1980s, considerable efforts have been undertaken by the polymer ageing community to develop non-empirical kinetic models for predicting the radio-thermal oxidation and the remaining lifetime of polymer insulation under normal operating conditions in NPPs. The main difficulty has been deriving a kinetic model from the radio-thermal oxidation mechanistic scheme, in which γ irradiation generates hydroperoxides (POOH) that thermally decompose to produce radicals which initiate new oxidation chains. Indeed, in the concerned ranges of the dose rate (typically between 10^−2^ and 10^−1^ Gy·h^−1^) and temperature (between 30 and 50 °C), polymer insulation is expected to perish because of oxidation both initiated by the polymer radiolysis and the thermal decomposition of POOH [6,7,8]. The pioneering researchers in this field were Gillen and Clough [9], who proposed a kinetic model for predicting the degradation of a PVC cable jacket that was exposed to radiation at a low ageing temperature in order to understand its surprisingly rapid ageing over 12 years under 0.25 Gy·h^−1^ at 43 °C. However, this model fails to predict the predominance of thermal ageing at very low dose rates.

Recently, it was shown that it is possible to derive a much more robust analytical solution from the radio-thermal oxidation mechanistic scheme without having to assume the thermal stability of POOH [8], a very common assumption that is responsible for serious inaccuracies in all other previous kinetic models. In particular, this new solution allowed researchers to successfully predict the global trends of the radio-thermal oxidation kinetics of Si-XLPE insulation from the molecular (i.e., the concentrations of oxidation products) up to macroscopic scale (lifetime), including the macromolecular scale (i.e., the concentration of elastically active chains).

However, in this kinetic model, the lifetime is always determined based on purely mechanical considerations, in particular when the polymer insulation almost completely loses its ability to prevent plastic deformation. That is the reason why the conventional end-of-life criterion used for cable application in the nuclear industry is a low value ($\varepsilon_{F}$) of the elongation at break ($\varepsilon_{R}$), typically: $\varepsilon_{R}=\varepsilon_{F}=50\%$ [10]. For Si-XLPE insulation, it was shown that this fracture criterion is reached at a very low conversion ratio during the chain scission process. In fact, it is related to a critical value ([POOH]_F_) of the hydroperoxide concentration, corresponding to the onset of the rapid auto-acceleration of the oxidation reaction when triggering the thermal initiation, typically: [POOH] = [POOH]_F_ ≈ 1.6 × 10^−1^ mol·L^−1^ [8].

In the context of an extension of the operating life of NPPs, the use of a purely mechanical criterion could pose a problem for cable life management. Indeed, several authors observed that the fracture properties generally degrade faster than several other functional properties of the polymer insulation, in particular its electrical properties [11,12,13,14,15,16]. In other words, fracture properties would lead to a significant underestimation of the lifetime of polymer insulation. That is the reason why the definition of a more relevant end-of-life criterion specific to the application of electrical cable insulation, i.e., of the dielectric type, is still a topical issue today [17,18,19]. However, taking into account the wide variety of electrical properties, what criterion could be used?

To date, two electrical properties closely related to the chemical structure of the polymer insulation have been the subject of several detailed analytical studies: the real part of permittivity $\varepsilon^{\prime }$ (defined as the “dielectric constant”) and volume resistivity R [20]. The literature compilations of the $\varepsilon^{\prime }$ and R values that have been reported for common polymers show that these two properties adversely evolve with the incorporation of oxygen atoms into the polymer structure [20,21,22,23,24].

In addition, it was often reported that oxidation leads to the formation and growth of dissipation bands in moderate frequency domains of the dielectric spectrum (typically, between 1 Hz and 10^5^ Hz at room temperature), where the polymer initially displays an almost ideal insulation behavior [20,24,25]. These bands are the manifestation of a physical phenomenon called “orientation polarization” or “Debye polarization”, occurring when groups of atoms have a permanent dipole moment [24]. This is particularly the case for polar oxygenated groups, such as carbonyls and hydroxyls, which are easily oriented in the direction of the applied electrical field. That is the reason why, in initially nonpolar polymers, such as low-density (LDPE) and crosslinked low-density polyethylene (XLPE), oxidation leads to a significant increase in the dielectric constant $\varepsilon^{\prime }$ from typically 2.3±0.3 up to values higher than 4.5 [14,26,27,28,29,30,31], whereas the volume resistivity R dramatically drops from typically 10^16±2^ Ω·cm to an asymptotic value of around 10^12±1^ Ω·cm [31,32,33,34,35,36].

Based on these careful analyses, two different routes can be proposed to predict $\varepsilon^{\prime }$ from the polymer structure:

- According to Lorentz [37] and Lorenz [38], $\varepsilon^{\prime }$ would be related to the molar polarization P of a given dielectric material as follows:
  (1) $$P=\frac{\varepsilon^{\prime }-1}{\varepsilon^{\prime }+2}V$$
  i.e.,
  (2) $$\varepsilon^{\prime }=\frac{1+2\left(P/V\right)}{1-\left(P/V\right)}$$
  where V is the molar volume. In addition, according to van Krevelen [20], P and V would obey molar additive rules, i.e., they could be calculated by summing the molar contributions of the different chemical groups composing the monomer unit or any other constitutive repeating unit (CRU):
  (3) $$P=\sum^{\;}P_{i}\;\mathrm{and}\;V=\sum^{\;}V_{i}$$
  As an example, Table 1 summarizes the values of $P_{i}$ and $V_{i}$ proposed by Goedhart [39] and Fedors [40] for methylene, but also for the various types of oxidation products generally detected during the radio-thermal ageing of PE. It should be noted that few of them were checked by van Krevelen in his handbook [20]. Thus, high uncertainties remain for many data (written in italics), in particular for ketones, carboxylic acids, hydroxyls and hydroperoxides.

- According to Darby [41], as electrical forces caused by polarizability and polar moment also determine the cohesive energy, a relationship should be expected between the dielectric constant $\varepsilon^{\prime }$ and the solubility parameter $\delta_{\mathrm{sol}}$. Based on a literature compilation of the $\varepsilon^{\prime }$ and $\delta_{\mathrm{sol}}$ values reported for common polymers, Darby proposed the following empirical proportionality:
(4) $$\delta_{\mathrm{sol}}\approx 7\varepsilon^{\prime }$$
  i.e.,
  (5) $$\varepsilon^{\prime }\approx 1.4\times 10^{-1}{\;\delta }_{\mathrm{sol}}$$
  It should be recalled that $\delta_{\mathrm{sol}}$ is related to the molar attraction constant F as follows:
  (6) $$\delta_{\mathrm{sol}}=\frac{F}{V}$$
  where, according to van Krevelen [20], F and V are molar additive functions:
  (7) $$F=\sum^{\;}F_{i}\;\mathrm{and}\;V=\sum^{\;}V_{i}$$

Values of $F_{i}$ proposed by Small [42], van Krevelen [43] and Hoy [44] for methylene and the various oxidation groups of PE are also listed in Table 1.

According to the literature, several other electrical properties could be deduced from $\varepsilon^{\prime }$ using empirical relationships. As an example, Cotts and Reyes [21] proposed the following power law for the dissipation factor $\tan\left(\delta_{\mathrm{el}}\right)$:

(8) $$\tan\left(\delta_{\mathrm{el}}\right)\approx {\left(\frac{\varepsilon^{\prime }}{10}\right)}^{5}$$

Knowing $\varepsilon^{\prime }$ and $\tan\left(\delta_{\mathrm{el}}\right)$, it would then be very easy to deduce the imaginary part of permittivity $\varepsilon^{\prime \prime }$ that is related to dielectric losses (which is, for this reason, commonly called the “dielectric absorption”) using its common definition:

(9) $$\varepsilon^{\prime \prime }=\tan\left(\delta_{\mathrm{el}}\right)\times \varepsilon^{\prime }$$

Its introduction into Equation (8) leads finally to:

(10) $$\varepsilon^{\prime \prime }\approx {\left(\frac{\varepsilon^{\prime }}{10}\right)}^{6}$$

However, the second electrical property of practical interest in the present study is the volume resistivity R. According to van Krevelen [20], it could be estimated using the following relationship:

(11) $${\mathrm{Log}}_{10}\left(R\right)=23-2\varepsilon^{\prime }$$

The objectives of the present article are twofold. On the one hand, the analytical model, recently developed for accurately describing the radio-thermal oxidation kinetics of Si-XLPE insulation [8], will be completed with several structure/property relationships that will additionally help to predict the changes in electrical properties. Our attention will first be focused on the dielectric constant because, as shown above, this electrical property can be directly related to the chemical structure, whereas the volume resistivity can be deduced from the dielectric constant. To achieve this first objective, the approach appearing to be by far the least empirical, i.e., starting from the research works of Lorentz [37] and Lorenz [38], should be used because the final kinetic model must be as efficient in simulation as in extrapolation. On the other hand, new end-of-life criteria specific for the industrial application under consideration, i.e., of the dielectric type, will be proposed for both the dielectric constant and the volume resistivity. The lifetimes determined based on electrical and mechanical considerations will then be compared in order to draw crucial conclusions for the electric utility companies.

## 2. Materials and Methods

### 2.1. Materials

Additive-free Si-XLPE films of about 500 µm thick were directly provided by Nexans NRC (Lyon, France). These films were produced through the extrusion of a linear low-density polyethylene (LDPE) and grafted with vinyl tri-methoxy silane side groups. The chemical crosslinking was then performed via immersion in water at 65 °C for 48 h [45]. The density, crystallinity ratio and gel content of the resulting Si-XLPE are about 0.914, 42.1% and 71%, respectively. The experimental procedures for determining these initial characteristics are detailed in Section 2.3, except for the gel fraction, which has already been reported in reference [8].

### 2.2. Radio-Thermal Ageing Conditions

Radio-thermal ageing was performed in the Panoza and Roza facilities at UJV Rez, Czech Republic, with a ^60^Co γ-ray source at different temperatures. All the exposure conditions are summarized in Table 2. It should be noted that the ageing experiments numbered 1, 3 and 4 were performed at three distinct dose rates (8.5, 77.8 and 400 Gy·h^−1^, respectively) at low temperature close to ambient in order to investigate the effect of dose rate on the oxidation kinetics. In contrast, the ageing experiments numbered 1 and 2 were performed at almost the same dose rate (8.5 and 6.0 Gy·h^−1^, respectively) but at two different temperatures (47 and 86 °C) in order to investigate the effect of temperature on the oxidation kinetics.

### 2.3. Experimental Characterizations

#### 2.3.1. Physico-Chemical Analyses

After ageing, the Si-XLPE films were characterized through FTIR spectroscopy in transmission mode in order to determine the exact nature and the relative proportion of the different oxidation products that can significantly affect the electrical properties. FTIR spectra were recorded from 4000 to 400 cm^−1^ with a Perkin Elmer FTIR Frontier spectrometer (Perkin Elmer, Villebon-sur-Yvette, France), after averaging the 16 scans that were taken at a resolution of 4 cm^−1^. For each film, at least three FTIR measurements were performed. However, because a large variety of hydroxyl and carbonyl products are formed during the radio-thermal ageing of Si-XLPE and their main IR absorption bands are often overlapped [46,47,48,49,50,51], FTIR spectroscopy was coupled with chemical gas derivatization, with ammonia (NH_3_) acting as the gaseous reagent. Indeed, NH_3_ is well known for transforming carboxylic acids into carboxylates, and esters and anhydrides into primary amides, thus inducing a significant shift of the IR absorption bands of these two carbonyl products along the wavenumber axis [49]. This chemical deconvolution method has been extensively detailed in the literature for linear PE, for instance in references [49,51].

**Figure 1:**
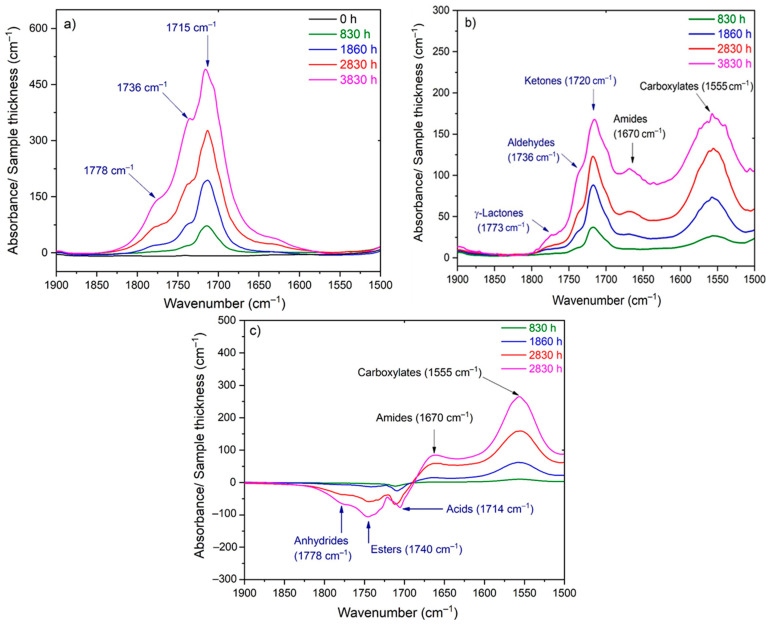
Changes in the carbonyl and carboxylate region of the FTIR spectrum of Si-XLPE during its radio-thermal ageing in air under 77.8 Gy·h^−1^ at 47 °C: (**a**) before NH_3_ treatment; (**b**) after NH_3_ treatment; (**c**) subtraction of the two previous spectra to evidence the consumption of carbonyls and the formation of carboxylates during the NH_3_ treatment.

As an example, Figure 1 shows the changes in the carbonyl and carboxylate region (i.e., typically between 1850 and 1500 cm^−1^) of the FTIR spectrum of Si-XLPE during its radio-thermal ageing in air under 77.8 Gy·h^−1^ at 47 °C (a) before and (b) after NH_3_ treatment. The subtraction of these two spectra, i.e., (c)=(b)−(a), facilitates the calculation of the carbonyl products consumed, including anhydrides (centered at about 1778 cm^−1^), linear esters (1740 cm^−1^) and carboxylic acids (1714 cm^−1^), and also the products formed during the NH_3_ treatment, including amides (1670 cm^−1^) and carboxylates (1555 cm^−1^). In addition, the FTIR spectrum after NH_3_ treatment (b) shows the carbonyl products that have not reacted with NH_3_, including cyclic esters (i.e., γ-lactones, centered at about 1773 cm^−1^), aldehydes (1736 cm^−1^) and ketones (1720 cm^−1^). The concentration of these different carbonyl products [P=O] was determined by applying the classical Beer–Lambert’s law:

(12) $$\left[\mathrm{Prod}\right]=\frac{\mathrm{OD}}{\mathrm{ep}\;\varepsilon }$$

where [Prod] is the concentration of the oxidation product under consideration (expressed in mol·L^−1^), OD is the optical density of its IR absorption band (dimensionless), ε is its molar extinction coefficient (L·mol^−1^·cm^−1^), and ep is the film thickness (cm).

The orders of magnitude of ε chosen for this calculation were determined in a previous publication [51], except for γ-lactones [52] and anhydrides [53]. They are recalled in Table 3.

**Figure 2:**
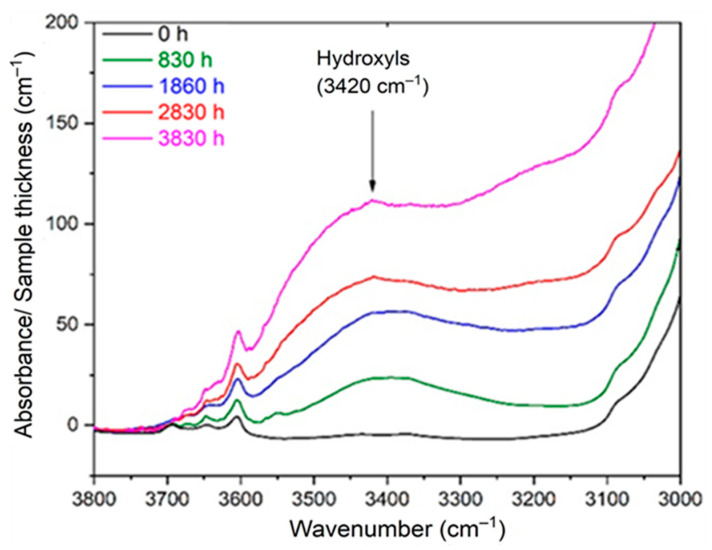
Changes in the hydroxyl region of the FTIR spectrum of Si-XLPE during its radio-thermal ageing in air under 77.8 Gy·h^−1^ at 47 °C.

As the hydroperoxides (POOH) were already titrated using differential scanning calorimetry (DSC) in a previous paper [8], the concentration of alcohols [P–OH] was also determined by applying Equation (12) to the composite IR absorption band of all the hydroxyl products located around 3420 cm^−1^ (see Figure 2). The corresponding molar extinction coefficient is also reported in Table 3. In fact, the concentration of alcohols was deduced from the total concentration of hydroxyls by subtracting the concentrations of hydroperoxides and carboxylic acids.

Due to the very wide variety of oxidation products and the lack of knowledge of structure/property relationships in this area (for instance, see Table 1), it is obvious that it is impossible to investigate the impact of each oxidation product on the electrical properties of Si-XLPE for the time being. In contrast, it seems more reasonable to limit the study to the impact of the main oxidation product.

In addition, it seems relevant to determine the total concentration of oxygen consumed by the oxidation reaction, namely the oxygen consumption ($Q_{O2}$), then to try to establish a relationship between this concentration and the changes in the electrical properties. As shown in Section 4, if making an assumption about the main oxidation product, oxygen consumption can be deduced from the changes in polymer density. Indeed, in the literature, it has repeatedly been reported that the incorporation of “heavy” atoms, such as oxygen, into a polymer structure initially containing many “light” atoms (i.e., carbon and hydrogen) induces an increase in its density [32,54,55,56,57,58].

The density of the Si-XLPE films was determined through hydrostatic weighing at room temperature (23 °C) with a Mettler Toledo MS104TS microbalance (Metler Toledo SAS, Viroflay, France). The films were first weighed in air, then in immersion in ethanol, and their density ρ was determined by applying Archimedes’ principle:

(13) ρ=mAirmAir−mImρEth

where $m_{\mathrm{Air}}$ and mIm are the sample weights in air and in immersion, respectively, and $\rho_{\mathrm{Eth}}$ is the density of ethanol at 23 °C ($\rho_{\mathrm{Eth}}=0.789$ [59]).

The changes in the crystalline morphology of the Si-XLPE films during their radio-thermal ageing were measured using differential scanning calorimetry (DSC) under pure nitrogen. DSC thermograms were recorded with a TA instruments DSC Q1000 calorimeter (TA Instruments, Guyancourt, France) that had beforehand been calibrated with an indium reference. Film samples with a mass ranging between 5 and 10 mg were placed in a closed standard aluminum pan to be analyzed at between −50 °C and 250 °C, with a heating rate of 10 °C·min^−1^ under a nitrogen flow of 50 mL·min^−1^.

**Figure 3:**
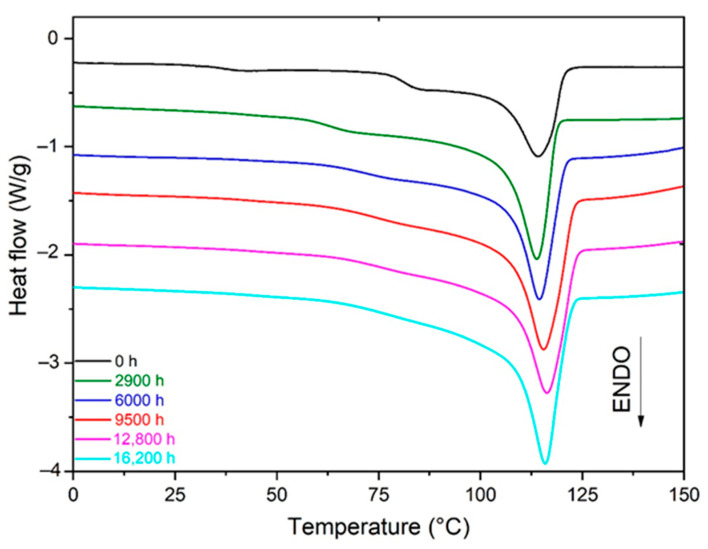
Changes in the melting peak of Si-XLPE during its radio-thermal ageing in air under 77.8 Gy·h^−1^ at 47 °C.

As an example, Figure 3 shows the changes in the DSC thermogram of the Si-XLPE during its radio-thermal ageing in air under 77.8 Gy·h^−1^ at 47 °C. As expected for a crosslinked polymer, the melting of the Si-XLPE occurred in a relatively wide temperature domain, typically ranged between 30 °C and 125 °C, with the maximum value of the main endothermic peak being located at around 114 °C. During the radio-thermal exposure, a gradual increase in the area under the main endothermic peak can be observed, thus indicating that an efficient chemi-crystallization process has occurred.

The crystallinity ratio $X_{C}$ of the Si-XLPE was determined with the common equation:

(14) $$X_{C}=\frac{\Delta H_{m}}{\Delta H_{m0}}\times 100$$

where $\Delta H_{m}$ is the sum of the areas under the endothermic peaks observed between 35 °C and 125 °C on the DSC thermogram, and $\Delta H_{m0}$ is the melting enthalpy of the PE crystal: $\Delta H_{m0}=$ 292 J·g^−1^ [60].

Then, the volume fraction of crystals $V_{C}$ was deduced from $X_{C}$ as follows:

(15) $$V_{C}=\frac{\rho }{\rho_{C}}X_{C}$$

where $\rho_{C}$ is the density of the crystalline phase of PE: $\rho_{C}=$ 1.014 [61].

#### 2.3.2. Electrical Measurements

After ageing, the Si-XLPE films were also characterized using two complementary electrical techniques in order to assess the impact of oxidation on the electrical properties. On the one hand, their complex relative permittivity $\varepsilon^{*}$ was determined through dielectric spectroscopy with a Novocontrol Alpha Dielectric analyzer v2.2 (Novocontrol Technologies, Montabaur, Germany). It should be recalled that $\varepsilon^{*}$ is described as follows:

(16) $$\varepsilon^{*}=\varepsilon^{\prime }-{j\varepsilon }^{\prime \prime }$$

where $\varepsilon^{\prime }$ is the real part of permittivity (defined as the “dielectric constant”) and $\varepsilon^{\prime \prime }$ is the imaginary part of permittivity related to the dielectric losses of the material (commonly called the “dielectric absorption”). Experimental tests were performed at room temperature with the following parameters: applied voltage = 3 Vrms; frequency range = 10^−2^–10^6^ Hz.

On the other hand, the volume resistivity of the Si-XLPE films was determined according to ASTM D257-14 [62]. Gold electrodes (sensing area ~3.14 cm^2^) were deposited on specimens using a plasma cold sputtering system. An electric field equal to 5 kV·mm^−1^ was applied through a Keithley 2290E-5 DC power supply (Keithley Instruments, Cleveland, Ohio, USA). The current was recorded through a Keysight B2980A (Keyseight Technologies, Santa Rosa, California, USA) and the volume resistivity R (expressed in Ω·cm was finally obtained through the following equation:

(17) $$R=\frac{1}{\sigma }=\frac{E}{J}$$

where σ is the electrical conductivity in S·cm^−1^, E is the applied electric field in V·cm^−1^ and J is the current density in A·cm^−2^.

## 3. Foundations of the Kinetic Model

The mechanistic scheme chosen for accurately describing the radio-thermal oxidation of Si-XLPE in the domain of practical interest for nuclear power plant operators (i.e., for 1.6 × 10^−7^ < I < 5.0 × 10^−1^ Gy·s^−1^) at a low temperature close to ambient has been detailed in previous publications [6,7,8]. As a reminder, the main feature of this mechanistic scheme is that oxidation is initiated by both the polymer radiolysis (1R) and the thermal decomposition of POOH in bimolecular mode (1T):

- Initiation:
  (1R) PH + hν → P^•^ + ½H_2_           ($r_{i}=10^{-7}\;G_{i}I$)
  (1T) 2POOH → P^•^ + PO_2_^•^           (k_1_)
- Propagation:
  (2) P^•^ + O_2_ → PO_2_^•^              (k_2_)
  (3) PO_2_^•^ + PH → POOH + P^•^         (k_3_)
- Termination:
  (4) P^•^ + P^•^ → Inactive products        (k_4_)
  (5) P^•^ + PO_2_^•^ → Inactive products      (k_5_)
  (6) PO_2_^•^ + PO_2_^•^ → Inactive products + O_2_    (k_6_)

where PH, POOH, P^•^ and PO_2_^•^ designate an oxidation site, an hydroperoxide, alkyl and peroxy radicals, respectively. δ, λ, and μ are stoichiometric coefficients. r_i_, G_i_ and k_j_ (with j = 1, …, 6) are the radiochemical initiation rate, the radical yield and the rate constants, respectively.

The system of differential equations derived from this mechanistic scheme was analytically solved using only two simplifying (but realistic) assumptions [8]:

1 Oxidation is mainly initiated by the polymer radiolysis that occurs throughout the exposure (i.e., $r_{i}\gg 2k_{1}{\left[\mathrm{POOH}\right]}^{2}$), with the thermal decomposition of POOH being an additional (but secondary) source of radicals for the long term;
2 The radical species reach a steady-state regime from the early periods of the radio-thermal exposure (i.e., d[Rad]/dt=0).

By only using these two assumptions, the following equations were found for:

- The concentration of POOH:
  (18) $$\left[\mathrm{POOH}\right]={\left[\mathrm{POOH}\right]}_{\infty }\frac{1-b\mathrm{Exp}\left(-\mathrm{Kt}\right)}{1+b\mathrm{Exp}\left(-\mathrm{Kt}\right)}$$
  with
  (19) $${\left[\mathrm{POOH}\right]}_{\infty }={\left(\frac{k_{3}\left[\mathrm{PH}\right]}{2k_{1b}}{\left(\frac{r_{i}}{2k_{6}}\right)}^{1/2}\frac{\beta C}{1+\beta C}\right)}^{1/2}$$
  (20) $$K=2{\left(2k_{3}\left[\mathrm{PH}\right]k_{1b}{\left(\frac{r_{i}}{2k_{6}}\right)}^{1/2}\frac{\beta C}{1+\beta C}\right)}^{1/2}$$
  and
  (21) $$b=\frac{{\left[\mathrm{POOH}\right]}_{\infty }-{\left[\mathrm{POOH}\right]}_{\mathrm{ini}}}{{\left[\mathrm{POOH}\right]}_{\infty }+{\left[\mathrm{POOH}\right]}_{\mathrm{ini}}}$$
  where [POOH]_ini_ and [POOH]_∞_ are the initial and steady concentrations of hydroperoxides, respectively. As for the weakly pre-oxidized samples, it is usually observed that: ${\left[\mathrm{POOH}\right]}_{\infty }\gg {\left[\mathrm{POOH}\right]}_{\mathrm{ini}}$ [51,63]. It can thus be considered that: b ≈ 1.

- The concentration of carbonyls:
  (22) $$\begin{array}{c} \left[P=O\right]=\left[\gamma_{1\mathrm{CO}}\frac{k_{3}\left[\mathrm{PH}\right]}{2}{\left(\frac{r_{i}}{2k_{6}}\right)}^{1/2}\frac{\beta C}{1+\beta C}+\gamma_{6\mathrm{CO}}\frac{r_{i}}{2}{\left(\frac{\beta C}{1+\beta C}\right)}^{2}\right]t\\ +2\gamma_{1\mathrm{CO}}\frac{k_{3}\left[\mathrm{PH}\right]}{K}{\left(\frac{r_{i}}{2k_{6}}\right)}^{1/2}\frac{\beta C}{1+\beta C}\left(\frac{1}{1+b\mathrm{Exp}\left(-\mathrm{Kt}\right)}-\frac{1}{1+b}\right) \end{array}\;\;$$
  where $\gamma_{1\mathrm{CO}}$ and $\gamma_{6\mathrm{CO}}$ are the respective formation yields of carbonyls in thermal initiation (1T) and termination (6).

- The oxygen consumption:

(23) $$Q_{O2}=\left[k_{3}\left[\mathrm{PH}\right]{\left(\frac{r_{i}}{2k_{6}}\right)}^{1/2}\frac{\beta C}{1+\beta C}+r_{i}\frac{\beta C}{1+\beta C}\left(1-\frac{\beta C}{2\left(1+\beta C\right)}\right)\right]t$$

In Equations (19), (20), (22) and (23), C is the oxygen concentration in the Si-XLPE films, which is related to the oxygen partial pressure P_O2_ in the exposure environment according to the classical Henry’s law:

(24) $$C=S\times P_{O2}$$

where S is the coefficient of oxygen solubility for the polymer. The typical values of S reported for low-density polyethylene (LDPE) in the literature are about 1.8 × 10^−8^ mol·L^−1^·Pa^−1^ regardless of the temperature [64]. As an example, in the case of an ageing in air under atmospheric pressure for which $P_{O2}=0.21\times 10^{5}\mathrm{Pa}$, Equation (24) finally leads to: $C=3.8\times 10^{-4}\;\mathrm{mol}\cdot L^{-1}$.

In addition, β^−1^ corresponds to the critical value of the oxygen concentration $C_{C}$ above which oxygen excess is reached:

(25) $$\beta =\frac{1}{C_{C}}\approx \frac{2k_{6}k_{2}}{k_{5}\left[k_{3}\left[\mathrm{PH}\right]+{\left(2r_{i}k_{6}\right)}^{1/2}\right]}$$

In a recent publication [8], it was shown that Equations (18) and (22) can be used for predicting the changes in the concentrations of hydroperoxides and carbonyls (for instance, carboxylic acids) in Si-XLPE insulation in air in different radio-thermal environments. As expected (see assumption (a)), a satisfying agreement was obtained between the theory and the experiments as long as thermal initiation (1T) remained a secondary source of radicals relative to radiochemical initiation (1R), i.e., under the three dose rates under study (from 8.5 to 400 Gy·h^−1^) at low temperatures close to ambient (i.e., 47 and 21 °C). However, a poorer agreement was obtained under the lowest dose rate (i.e., 6.0 Gy·h^−1^) at the highest temperature (86 °C) because, in these critical radio-thermal exposure conditions, thermal initiation becomes of the same order of magnitude as (if not greater than) the radiochemical initiation. For context, the values of the different kinetic parameters used for these simulations have been recalled in Table 4.

## 4. Main Oxidation Products

Table 5 summarizes the relative proportions of the different oxidation products measured during the radio-thermal ageing of the Si-XLPE with FTIR spectroscopy coupled with chemical gas derivatization and DSC. As already reported by many authors in the literature for other types of PE, for instance in references [48,51,65,66,67,68], ketones are the main carbonyl products in Si-XLPE. It is noteworthy that ketones also occur in higher concentrations than hydroxyl products, in particular hydroperoxides (POOH). This is even more obvious when γ irradiation is performed at a high temperature (i.e., 86 °C) because POOH thermally decompose and thus become hardly detectable through DSC. In fact, their concentration is of the order of magnitude of the DSC detection threshold, which is around 10^−3^ L·mol^−1^ [51].

For these reasons, as a first approach, this study will be limited to investigating the impact of ketones on the electrical properties of Si-XLPE. Of course, before being applied, this assumption will first be checked against another important physico-chemical property closely related to oxygen consumption in the next section: the polymer density.

## 5. Calculation of the Changes in Density

First of all, it should be recalled that the density of a semi-crystalline polymer can be expressed as a function of the densities of its amorphous ($\rho_{a}$) and crystalline phases ($\rho_{C}$):

(26) $$\rho =V_{C}\rho_{C}+\left(1-V_{C}\right)\rho_{a}$$

where $V_{C}$ is the volume fraction of crystals.

According to Equation (26), two main causes can be responsible for an increase in ρ during radio-thermal ageing:

- As recalled in Section 2.3, the incorporation of “heavy” atoms, such as oxygen, into a polymer structure initially containing many “light” atoms (i.e., carbon and hydrogen) induces an increase in its density [32,54,55,56,57,58]. Since crystals are impermeable to oxygen, oxidation only occurs in the amorphous phase where it thus induces an increase in $\rho_{a}$.
- In Si-XLPE, oxidation leads to a predominance of chain scissions over crosslinking [8]. Chain scissions progressively destroy the macromolecular network from which short linear fragments are extracted, which can easily migrate towards crystalline lamellae when the amorphous phase is in a rubbery state. The integration of these short fragments with crystalline lamellae induces a chemi-crystallization, i.e., a thickening of crystalline lamellae and an increase in the crystallinity ratios (i.e., $X_{C}$ and $V_{C}$).

The resulting changes in ρ can be thus written as follows:

(27) $$d\rho ={\frac{\partial \rho }{\partial \rho_{a}}|}_{V_{C}=V_{C\;\mathrm{ini}}}{d\rho }_{a}+{\frac{\partial \rho }{\partial V_{C}}|}_{\rho_{a}=\rho_{a\;\mathrm{ini}}}{\mathrm{dV}}_{C}$$

where $V_{C\;\mathrm{ini}}$ and $\rho_{a\;\mathrm{ini}}$ are the respective values of $V_{C}$ and $\rho_{a}$ for the unoxidized polymer. For Si-XLPE, $V_{C\;\mathrm{ini}}=37.9\%$ and $\rho_{a\;\mathrm{ini}}=$ 0.85 [69].

Considering Equation (26), it can be easily shown that:

(28) $${\frac{\partial \rho }{\partial \rho_{a}}|}_{V_{C}=V_{C\;\mathrm{ini}}}=1-V_{C\;\mathrm{ini}}\;\mathrm{and}\;{\frac{\partial \rho }{\partial V_{C}}|}_{\rho_{a}=\rho_{a\;\mathrm{ini}}}=\rho_{C}-\rho_{a\;\mathrm{ini}}$$

The introduction of these two quantities into Equation (27) gives:

(29) $$d\rho =\left(1-V_{C\;\mathrm{ini}}\right){d\rho }_{a}+\left(\rho_{C}-\rho_{a\;\mathrm{ini}}\right){\mathrm{dV}}_{C}$$

i.e.,

(30) $$\Delta \rho =\left(1-V_{C\;\mathrm{ini}}\right)\Delta \rho_{a}+\left(\rho_{C}-\rho_{a\;\mathrm{ini}}\right)\Delta V_{C}$$

As the final objective is to relate the changes in the density of the Si-XLPE (ρ) to its oxygen consumption ($Q_{O2}$) during the radio-thermal ageing, this can be written as:

(31) $$\frac{\Delta \rho }{\Delta Q_{O2}}=\left(1-V_{C\;\mathrm{ini}}\right)\frac{\Delta \rho_{a}}{\Delta Q_{O2}}+\left(\rho_{C}-\rho_{a\;\mathrm{ini}}\right)\frac{\Delta V_{C}}{\Delta Q_{O2}}$$

The final challenge was to determine the values of the two ratios, $\Delta \rho_{a}/\Delta Q_{O2}$ and $\Delta V_{C}/\Delta Q_{O2}$.

The first ratio $\Delta \rho_{a}/\Delta Q_{O2}$ was assessed using a structure–property relationship that has previously been established in literature. According to Pascault et al. [70], $\rho_{a}$ depends on the atomic composition, which can be represented by a simple quantity, the “average atomic mass” $M_{a}$, which is determined from the monomer unit or any other constitutive repeating unit (CRU) as follows:

(32) $$M_{a}=\frac{M_{\mathrm{CRU}}}{N_{\mathrm{CRU}}}$$

where N_CRU_ and M_CRU_ are the total number of atoms and the molar mass of the CRU, respectively. As an example, for unoxidized Si-XLPE: $M_{\mathrm{CRU}\;\mathrm{ini}}=28\;g\cdot {\mathrm{mol}}^{-1}$ and $N_{\mathrm{CRU}\;\mathrm{ini}}=6$, so that: $M_{a\;\mathrm{ini}}=4.67\;g\cdot {\mathrm{mol}}^{-1}$.

Based on a literature compilation of the $\rho_{a}$ values reported for common amorphous and semi-crystalline polymers, they found that $\rho_{a}$ is an increasing linear function of $M_{a}$. Langlois et al. [55] tried to generalize this relationship with semi-crystalline polymers, in particular when the contribution of chemi-crystallization is negligible. For a radiation crosslinked low-density polyethylene (XLPE) with an initial volume fraction of crystals: $V_{C\;\mathrm{ini}}=51.4\%$, they found that:

(33) $$\frac{\Delta \rho }{\Delta M_{a}}=0.125\pm 0.025\mathrm{mol}\cdot {\mathrm{cm}}^{-3}$$

Let us recall that, when chemi-crystallization is negligible, it can be written as:

(34) $$\frac{\Delta \rho_{a}}{\Delta M_{a}}=\frac{1}{1-V_{C\;\mathrm{ini}}}\frac{\Delta \rho }{\Delta M_{a}}$$

It can thus finally be written as:

(35) $$\frac{\Delta \rho_{a}}{\Delta M_{a}}=0.257\pm 0.052\mathrm{mol}\cdot {\mathrm{cm}}^{-3}$$

Applying Equation (35) requires knowing under what major structure oxygen is when it is chemically bonded to macromolecules: is it hydroperoxide, alcohol, ketone, aldehyde, carboxylic acid or ester? For each of these oxidation products, the oxidized polymer can simply be described by using a CRU containing p carbon atoms (with p≥2), as shown in Table 6. In each case, several quantities can be calculated, in particular the molar mass ($M_{\mathrm{CRU}})$ and the total number of atoms of the CRU ($N_{\mathrm{CRU}})$, and the number of O_2_ molecules chemically consumed per carbon atom ($n_{O2}$). From these three quantities, two key ratios can be deduced in turn: ${\Delta M}_{a}/{\Delta n}_{O2}$ then ${\Delta M}_{a}/Q_{O2}$. The calculation of these different properties has been detailed in Appendix A for when hydroperoxides are the main oxidation products (i.e., for hydroperoxidized PE). This calculation can easily be generalized to all other oxidation products. The corresponding results are reported in Table 6.

Finally, the ratio $\Delta \rho_{a}/\Delta Q_{O2}$ was simply deduced as follows:

(36) $$\frac{\Delta \rho_{a}}{\Delta Q_{O2}}=\frac{\Delta \rho_{a}}{\Delta M_{a}}\times \frac{\Delta M_{a}}{\Delta Q_{O2}}$$

This last key ratio was calculated for all oxidation products, choosing the upper limit of the variation interval proposed by Langlois et al. (see Equation (35)) as the value for $\Delta \rho_{a}/\Delta M_{a}$, i.e., taking:

(37) $$\frac{\Delta \rho_{a}}{\Delta M_{a}}\approx 0.30\;\mathrm{mol}\cdot {\mathrm{cm}}^{-3}$$

Here again, the corresponding results are reported in Table 6 (in the last column).

**Figure 4:**
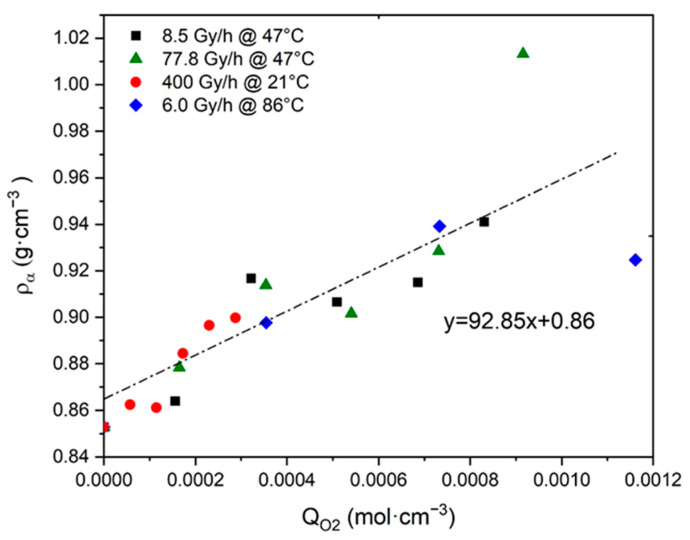
Density of amorphous phase versus oxygen consumption for Si-XLPE aged in the various radio-thermal environments under study.

The ratio $\Delta \rho_{a}/\Delta Q_{O2}$ was used to identify the major structure under which oxygen is chemically bonded to Si-XLPE macromolecules. In particular, $\rho_{a}$ was calculated with Equation (26) from the values of ρ and $V_{C}$ that were measured on the Si-XLPE films before and after their exposure to the different radiochemical environments under study. Then, $\rho_{a}$ was plotted in Figure 4 as a function of the values of $Q_{O2}$, which was previously calculated with Equation (23) for the same exposure conditions in reference [8]. Figure 4 clearly shows a master curve with an almost linear shape whose slope gives direct access to the ratio under investigation. The high value of the slope indicates that ketones would be the main oxidation products in Si-XLPE, as already found by FTIR spectroscopy coupled with chemical gas derivatization and DSC in Section 4. That is the reason why, in the present study, the ratio $\Delta \rho_{a}/\Delta Q_{O2}$ was set at:

(38) $$\frac{\Delta \rho_{a}}{\Delta Q_{O2}}\approx 70.27\;g\cdot {\mathrm{mol}}^{-1}$$

**Figure 5:**
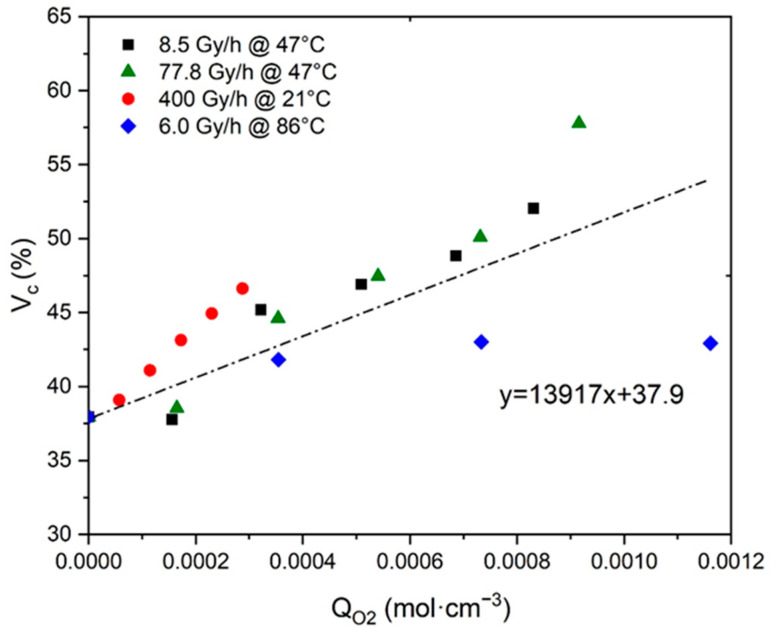
Volume fraction of crystals versus oxygen consumption for Si-XLPE aged in the various radio-thermal environments under study.

In contrast, the second ratio $\Delta V_{C}/\Delta Q_{O2}$ was directly assessed by plotting $V_{C}$ as a function of $Q_{O2}$ in Figure 5. Here again, Figure 5 highlights a master curve with an almost linear shape whose slope gives direct access to the ratio under investigation:

(39) $$\frac{\Delta V_{C}}{\Delta Q_{O2}}\approx 139.17{\;\mathrm{cm}}^{3}\cdot {\mathrm{mol}}^{-1}$$

The values found for the two ratios $\Delta \rho_{a}/\Delta Q_{O2}$ and $\Delta V_{C}/\Delta Q_{O2}$ were then introduced into Equation (31). Recalling that $V_{C\;\mathrm{ini}}=37.9\%$ and $\rho_{a\;\mathrm{ini}}=$ 0.85 for Si-XLPE, this can finally be written as:

(40) $$\frac{\Delta \rho }{\Delta Q_{O2}}=0.621\times 70.27+0.164\times 139.17$$

i.e.,

(41) $$\frac{\Delta \rho }{\Delta Q_{O2}}=66.46\;g\cdot {\mathrm{mol}}^{-1}$$

**Figure 6:**
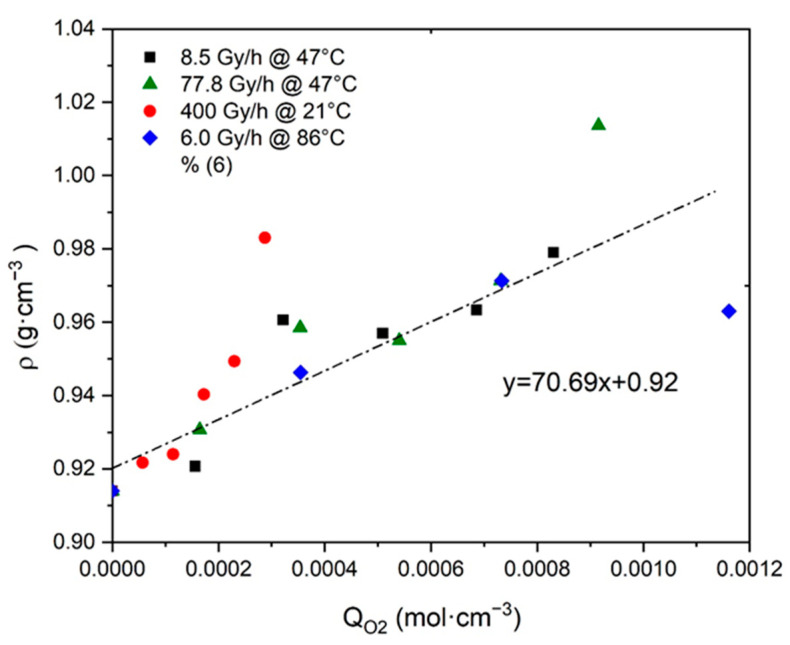
Density versus oxygen consumption for Si-XLPE aged in the various radio-thermal environments under study.

The reliability of this third ratio was checked by plotting ρ as a function of $Q_{O2}$ in Figure 6. It can be noted that the resulting master curve displays a slope value, which is of the same order of magnitude as the result of Equation (41). Confirming this allowed us to definitively validate the assumption that the density ρ of Si-XLPE would be a measurement of its oxygen consumption $Q_{O2}$.

## 6. Prediction of Electrical Properties

As shown in the previous section, ketones are the main oxidation products in Si-XLPE. The corresponding CRU is shown in Table 6. Let us recall that oxygen consumption $Q_{O2}$ can be written as:

(42) $$Q_{O2}=\frac{\rho_{a\;\mathrm{ini}}}{32}\frac{\Delta M_{\mathrm{UCR}}}{M_{\mathrm{UCR}\;\mathrm{ini}}}$$

where $M_{\mathrm{UCR}\;\mathrm{ini}}$ and $\rho_{a\;\mathrm{ini}}$ are the molar mass of the CRU ($M_{\mathrm{UCR}\;\mathrm{ini}}=14p$) and the density of the amorphous phase of the unoxidized Si-XLPE ($\rho_{a\;\mathrm{ini}}=0.85$ [69]), respectively.

Applying Equation (42) to ketones gives:

(43) $$Q_{O2}=\frac{\rho_{a\;\mathrm{ini}}}{16}n_{O2}$$

i.e., if Q_O2_ is expressed in mol·L^−1^:

(44) $$n_{O2}=1.9\times 10^{-2}\;Q_{O2}$$

Based on the CRU of the oxidized Si-XLPE, the molar additive rules reported for the molar polarization P and molar volume V in Equation (3) can be rewritten as follows:

(45) $$P=\left(p-1\right)P_{\mathrm{CH}2}+P_{\mathrm{CO}}$$

and

(46) $$V=\left(p-1\right)V_{\mathrm{CH}2}+V_{\mathrm{CO}}$$

where $P_{\mathrm{CH}2}$, $V_{\mathrm{CH}2}$, $P_{\mathrm{CO}}$ and $V_{\mathrm{CO}}$ are the molar contributions of the methylene and ketone groups to P and V, respectively. As a reminder, their values are summarized in Table 1. Thus, the ratio P/V can be written as:

(47) $$\frac{P}{V}=\frac{\left(p-1\right)P_{\mathrm{CH}2}+P_{\mathrm{CO}}}{\left(p-1\right)V_{\mathrm{CH}2}+V_{\mathrm{CO}}}$$

Knowing that, for ketones (see Table 6):

(48) $$n_{O2}=\frac{1}{2p}$$

it was finally obtained:

(49) $$\frac{P}{V}=\frac{P_{\mathrm{CH}2}+2n_{O2}\left(P_{\mathrm{CO}}-P_{\mathrm{CH}2}\right)}{V_{\mathrm{CH}2}+2n_{O2}\left(V_{\mathrm{CO}}-V_{\mathrm{CH}2}\right)}$$

i.e.,

(50) $$\frac{P}{V}=\frac{P_{\mathrm{CH}2}+3.8\times 10^{-2}\;Q_{O2}\left(P_{\mathrm{CO}}-P_{\mathrm{CH}2}\right)}{V_{\mathrm{CH}2}+3.8\times 10^{-2}\;Q_{O2}\left(V_{\mathrm{CO}}-V_{\mathrm{CH}2}\right)}$$

Knowing the ratio P/V, the Lorentz and Lorenz equation [37,38] was used to calculate the dielectric constant $\varepsilon^{\prime }$. This equation is recalled below:

(51) $$\varepsilon^{\prime }=\frac{1+2\left(P/V\right)}{1-\left(P/V\right)}$$

Let us note that, in the absence of ketones, this equation reveals the order of magnitude of the dielectric constant of unoxidized PE, i.e., $\varepsilon_{\mathrm{ini}}^{\prime }=2.3$ [20,21,23,24].

**Figure 7:**
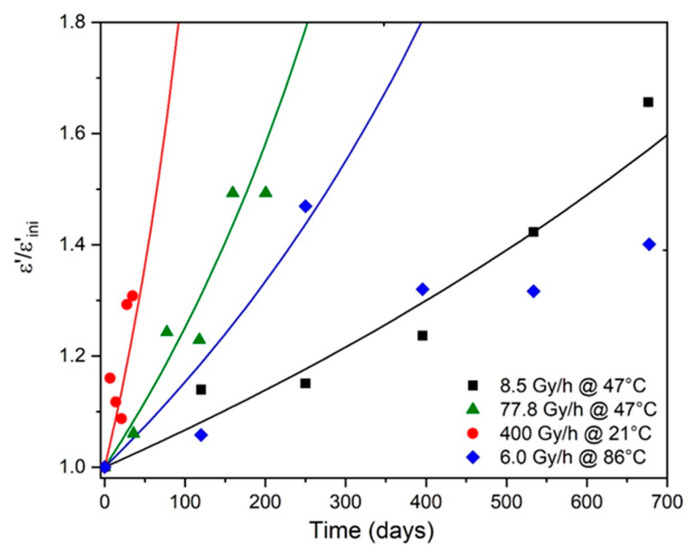
Changes in the dielectric constant (normalized by its initial value $\varepsilon_{\mathrm{ini}}^{\prime }$) of Si-XLPE in the various radio-thermal environments under study. Comparison between simulation with Equation (50) (solid lines) and experimental data (symbols).

The changes in $\varepsilon^{\prime }$ for the Si-XLPE films during their radio-thermal ageing were determined from the values of $Q_{O2}$ previously calculated with Equation (23) for the same exposure conditions as in reference [8]. However, it was rapidly concluded that it was impossible to use the value reported in Table 1 for $P_{\mathrm{CO}}$, because this largely underestimated $\varepsilon^{\prime }$ regardless of the exposure conditions. The value of $P_{\mathrm{CO}}$ that gave the best simulations for all the experimental data was finally chosen as: $P_{\mathrm{CO}}\approx 80{\;\mathrm{cm}}^{3}\cdot {\mathrm{mol}}^{-1}$. In contrast, all the other molar contributions were set according to the literature (see Table 1). The simulations obtained with Equation (51) for all the radio-thermal environments under study are shown in Figure 7.

As already explained in Section 3, a satisfying agreement between the theory and the experiments can be observed as long as the foundations of the kinetic model are checked, i.e., under the three dose rates under study (from 8.5 to 400 Gy·h^−1^) at low temperatures close to ambient (i.e., 47 and 21 °C). However, a poorer agreement is obtained under the lowest dose rate (i.e., 6.0 Gy·h^−1^) at the highest temperature (86 °C) because, in these critical radio-thermal exposure conditions, thermal initiation becomes of the same order of magnitude as (if not greater than) the radiochemical initiation.

**Figure 8:**
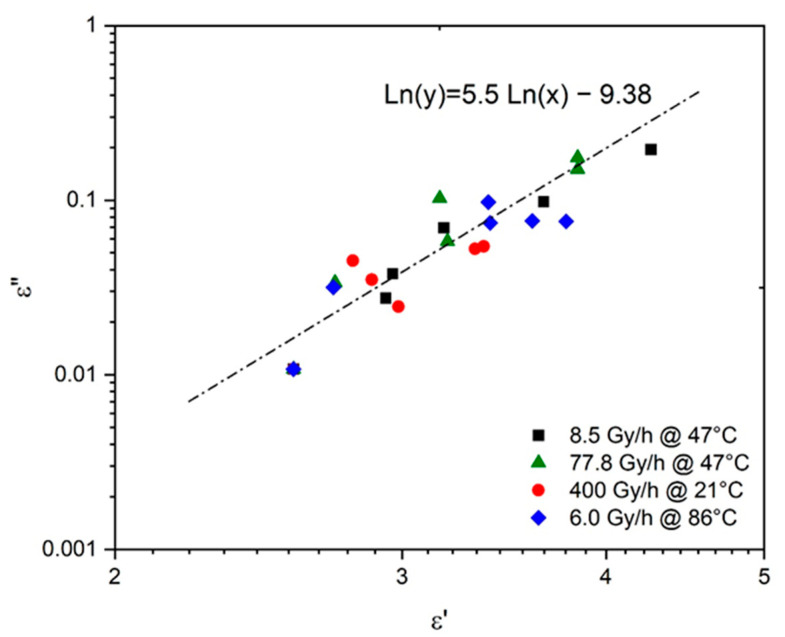
Dielectric absorption versus dielectric constant for Si-XLPE aged in the various radio-thermal environments under study.

The possibility of deducing the changes in $\varepsilon^{\prime \prime }$ from the simulations obtained in Figure 8 for $\varepsilon^{\prime }$ was carefully investigated. In particular, $\varepsilon^{\prime \prime }$ was plotted as a function of $\varepsilon^{\prime }$ in logarithm–logarithm coordinates in Figure 8. The following empirical relationship was found for oxidized Si-XLPE:

(52) $$\varepsilon^{\prime \prime }\approx {\left(\frac{\varepsilon^{\prime }}{5.5}\right)}^{5.5}$$

It should be noted that this Equation (52) is very close to Equation (10), previously determined by Cotts and Reyes [21] for common polymers.

**Figure 9:**
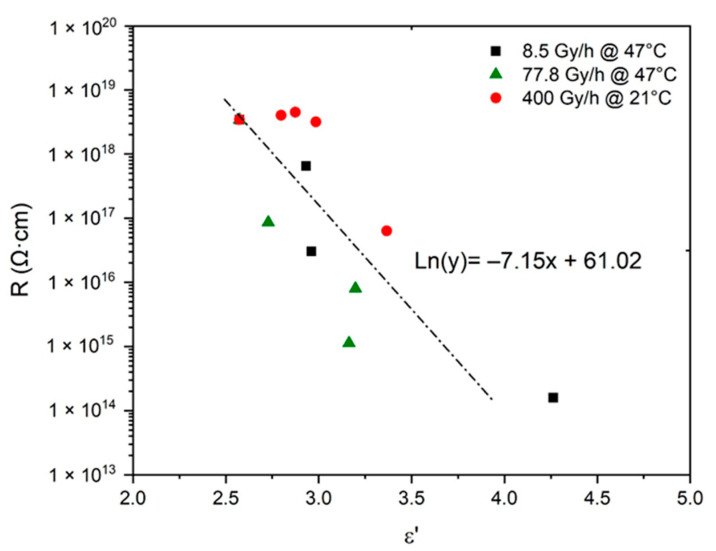
Volume resistivity versus dielectric constant for Si-XLPE aged in the various radio-thermal environments under study.

In addition, the possibility of deducing the changes in R from the simulations obtained in Figure 7 for $\varepsilon^{\prime }$ was also carefully investigated. R was plotted as a function of $\varepsilon^{\prime }$ in logarithm–linear coordinates in Figure 9. The following empirical relationship was found for oxidized Si-XLPE:

(53) $${\mathrm{Log}}_{10}\left(R\right)=26.5-3.1\varepsilon^{\prime }$$

Here again, it should be noted that Equation (53) is very close to Equation (11), previously determined by van Krevelen [20] for common polymers.

## 7. Proposal of an End-of-Life Criterion

**Figure 10:**
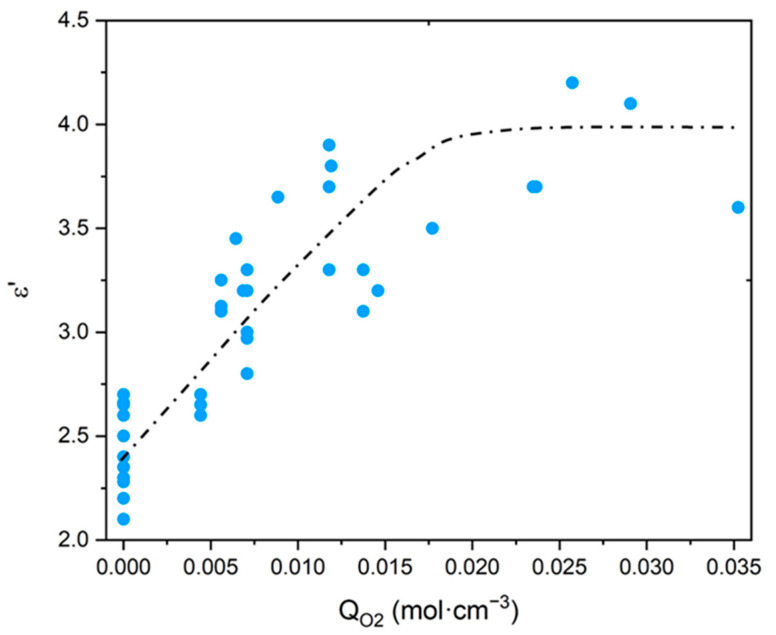
Dielectric constant versus oxygen concentration for polymers containing only C, H and O atoms in their CRU [20,21,22,23,24].

**Figure 11:**
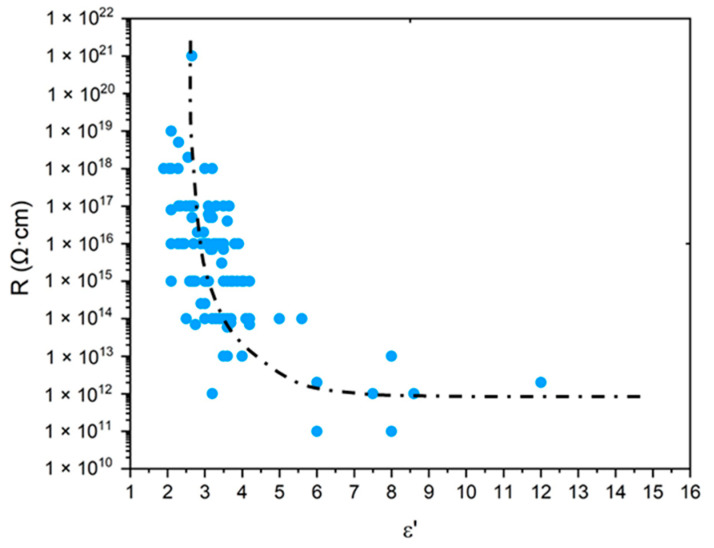
Volume resistivity versus dielectric constant for common polymers [20,21,22,23,24].

As explained in the introduction, only purely mechanical considerations are currently used for lifetime prediction, which poses a serious issue for cable life management in NPPs. The search for a more relevant end-of-life criterion, i.e., of the dielectric type, requires a careful analysis of the changes in the electrical properties of polymer structures. For this reason, the values of $\varepsilon^{\prime }$ and R of common polymers were compiled from the literature [20,21,22,23,24], then $\varepsilon^{\prime }$ was plotted as a function of the oxygen concentration in the CRU (i.e., $Q_{O2}$) in Figure 10, whereas R was plotted as a function of $\varepsilon^{\prime }$ in Figure 11. It can be observed that, for the polymers containing only C, H and O atoms in their CRU, $\varepsilon^{\prime }$ progressively increases with $Q_{O2}$ from typically 2.4±0.3 up to an asymptotic value of around 4.0. In the meantime, for all common polymers, R dramatically drops with $\varepsilon^{\prime }$ from typically $10^{17\pm 2}\;\Omega \cdot \mathrm{cm}$ to an asymptotic value of around $10^{12\pm 1}\;\Omega \cdot \mathrm{cm}$.

Thus, for values of $Q_{O2}$ higher than typically $1.8\times 10^{-3}\mathrm{mol}\cdot {\mathrm{cm}}^{-3}$, it is found that common polymers would exhibit the same electrical performance as glass, a material with poor insulating properties. As an example, for SiO_2_, the following values of $\varepsilon^{\prime }$ and R were reported in the literature: $\varepsilon^{\prime }\approx 3.9$ [71] and $R\approx 10^{12}\;\Omega \cdot \mathrm{cm}$ [72]. These are indeed of the same order of magnitude as the previous asymptotic values.

Regarding the Si-XLPE insulation under study, in Figure 6, it can be seen that the most aged samples have not yet reached this boundary behavior, but they are approaching it dangerously. Indeed, they are characterized by a maximum value of $Q_{O2}$ of about $1.2\times 10^{-3}\mathrm{mol}\cdot {\mathrm{cm}}^{-3}$.

Thereafter, it will be considered that R is dangerously approaching its asymptote when it is above a critical value of $\varepsilon^{\prime }$ of the order of $\varepsilon^{\prime }=\varepsilon_{F}^{\prime }\approx 3.5$. If normalized with respect to the dielectric constant of a nonpolar polymer, such as PE and its copolymers, this critical value can be rewritten as: $\varepsilon_{F}^{\prime }/\varepsilon_{\mathrm{ini}}^{\prime }\approx 1.5$.

Taking, as a first approach, $\varepsilon_{F}^{\prime }/\varepsilon_{\mathrm{ini}}^{\prime }\approx 1.5$ as the end-of-life criterion for Si-XLPE insulation, the lifetime $t_{F}\left(\varepsilon^{\prime }\right)$ can be graphically determined as it is in Figure 7 for the different radio-thermal environments under study. In Table 7, the values obtained for $t_{F}(\varepsilon^{\prime })$ are compared to those previously calculated with the conventional mechanical end-of-life criterion (i.e., when the elongation at break $\varepsilon_{R}$ reaches its critical value $\varepsilon_{F}=50\%$) for the same exposure conditions as in reference [8].

It is found that $t_{F}\left(\varepsilon^{\prime }\right)\geq 2{\;t}_{F}\left(\varepsilon_{R}\right)$ regardless of the exposure conditions, thus confirming the previous literature studies that have shown that the fracture properties degrade faster than electrical properties [11,12,13,14,15,16]. This result could be explained by considering the choice of the failure criterion for mechanical and electrical tests. Indeed, mechanical failure is considered to have been reached for a still fairly high value of $\varepsilon_{R}$ because, below this critical value, the cable cannot withstand a loss of coolant accident (LOCA), although it is still able to operate under normal conditions. In contrast, the critical value proposed here for $\varepsilon^{\prime }$, corresponding to the asymptotic value of the insulation resistivity beyond which the polymer is no longer a good insulator, really represents the end point of cable life because, above this value, the cable is no longer able to operate even under normal conditions. Thus, this result confirms the urgent need for a dielectric end-of-life criteria consistent with the mechanical behavior to predict the lifetime of electrical cable insulation in nuclear industry.

## 8. Conclusions

The analytical model, recently developed for accurately describing the radio-thermal oxidation kinetics of unfilled and unstabilized Si-XLPE insulation in NPPs [8], has been completed with several structure–property relationships in order to additionally predict the changes in its electrical properties. This model was derived from a mechanistic scheme in which the oxidation reaction is initiated both by the polymer radiolysis and the thermal decomposition of hydroperoxides, without making the usual assumption concerning the thermal stability of hydroperoxides. After an initial period where the oxidation kinetics occur at a constant rate, it also allows predicting the auto-acceleration of the oxidation kinetics when hydroperoxide decomposition is no longer negligible. Assuming that ketones are the main oxidation products in Si-XLPE, the model also calculates the oxygen consumption $Q_{O2}$ from which the changes in density ρ and dielectric constant $\varepsilon^{\prime }$ can be deduced. The validity of this assumption was first checked with FTIR spectroscopy coupled with chemical gas derivatization and DSC, then confirmed by density measurements.

Several other electrical properties can also be deduced from $\varepsilon^{\prime }$ using empirical relationships, such as the dielectric absorption $\varepsilon^{\prime \prime }$ or the volume resistivity R. From the changes in R with $\varepsilon^{\prime }$ for common polymers, a dielectric end-of-life criterion has been proposed to calculate the lifetime of Si-XLPE insulation in nuclear environments. The corresponding lifetime was found to be at least two times higher than that previously calculated with the conventional mechanical end-of-life criterion for the same exposure conditions as in reference [8]. This result, which can be explained by two different choices of end-of-life point, confirms the urgent need for dielectric end-of-life criteria consistent with the mechanical behavior to predict the lifetime of electrical cable insulation in nuclear industry.

Despite its apparent success, this multiscale approach for lifetime prediction needs to be improved in several places. First of all, the chemi-crystallization kinetics (i.e., the changes in crystallinity ratios $X_{C}$ and $V_{C}$) and its consequences for Si-XLPE density should be carefully and accurately analyzed so that it can be non-empirically modeled. To this end, the experimental results obtained from differential scanning calorimetry (DSC) and X-ray diffraction at both large and small angles (WAXS and SAXS) will be published in the coming months. In addition, the value of the molar polarization proposed in this study for ketones should be theoretically confirmed, e.g., from molecular dynamics calculations. These calculations will also allow us to check whether it is correct to only consider the main oxidation products (i.e., ketones) or whether other types of polar products (such as carboxylic acids, aldehydes and hydroperoxides), although in slightly lower concentration in Si-XLPE, should also be considered to more rigorously predict the changes in the dielectric constant. Finally, a wide field of research opens for the prediction of all the other electrical properties. There is no doubt that these prospects will constitute the challenges of many future publications.

## Figures and Tables

**Table 1:** Molar contributions to P, F and V of the methylene group and the various oxidation groups formed in PE [39,40,42,43,44]. Values written in italics are subject to high uncertainties.

Group	P_i_ (cm^3^·mol^−1^)	F_i_ (J^1/2^·cm^3/2^·mol^−1^)	V_i_ (cm^3^·mol^−1^)
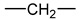	4.7	275	16.1
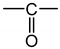	*10*	560	10.8
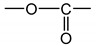	15	590	18
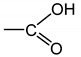	*15.8*	825	28.5
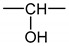	*9.6*	725	9
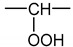	*14.8*	925	12.8

**Table 2:** Radio-thermal ageing conditions.

Ageing No.	Dose Rate (Gy·h^−1^)	Dose Rate (Gy·s^−1^)	Temperature (°C)	Maximum Duration (h)	Maximum Dose (kGy)
1	8.5	2.36 × 10^−3^	47	12,800	109
2	6.0	1.67 × 10^−3^	86	16,267	98
3	77.8	2.16 × 10^−2^	47	3830	298
4	400	1.11 × 10^−1^	21	668	269

**Table 3:** Wavenumbers and molar extinction coefficients [51,52,53] of the main oxidation products in Si-XLPE.

Oxidation Products	ν (cm^−1^)	ε (L·mol^−1^·cm^−1^)	Reference for ε
Hydroxyls	3420	70	[51]
Anhydrides	1778	730	[53]
γ-Lactones	1773	720	[52]
Esters	1740	590	[51]
Aldehydes	1736	270	[51]
Ketones	1720	300	[51]

**Table 4:** Values of the kinetic parameters used for modeling the oxidation kinetics of Si-XLPE in the various radio-thermal environments under study [8].

**T (°C)**	21	47	47	86
**I (Gy·h^−1^)**	400	77.8	8.5	6.0
**G_i_**	8	8	8	8
**k_1b_ (L·mol^−1^·s^−1^)**	5.0 × 10^−9^	2.5 × 10^−7^	2.4 × 10^−7^	1.0 × 10^−5^
**k_2_ (L·mol^−1^·s^−1^)**	10^8^	10^8^	10^8^	10^8^
**k_3_ (L·mol^−1^·s^−1^)**	1.6 × 10^−3^	1.9 × 10^−2^	1.9 × 10^−2^	3.6 × 10^−1^
**k_4_ (L·mol^−1^·s^−1^)**	8.0 × 10^11^	8.0 × 10^11^	8.0 × 10^11^	8.0 × 10^11^
**k_5_ (L·mol^−1^·s^−1^)**	1.2 × 10^10^	7.0 × 10^10^	9.0 × 10^10^	2.4 × 10^11^
**k_6_ (L·mol^−1^·s^−1^)**	5.0 × 10^4^	1.0 × 10^6^	2.0 × 10^6^	6.0 × 10^7^
**γ_1CO_ (%)**	90	70	75	100
**γ_6CO_ (%)**	90	70	75	100

**Table 5:** Relative proportions (expressed in mol%) of the different oxidation products in the Si-XLPE aged in the various radio-thermal environments under study.

**T (°C)**	21	47	47	86
**I (Gy·h^−1^)**	400	77.8	8.5	6.0
**[POOH] (mol%)**	16.2	19.9	15.9	–
**[Alcohols] (mol%)**	7.3	4.0	6.6	2.7
**[Anhydrides] (mol%)**	1.5	1.9	0.9	3.7
**[γ-Lactones] (mol%)**	1.0	1.5	2.1	3.0
**[Linear esters] (mol%)**	4.5	3.7	3.1	16.7
**[Aldehydes] (mol%)**	11.5	14.4	15.4	25.1
**[Ketones] (mol%)**	35.3	30.2	37.6	31.0
**[Carboxylic acids] (mol%)**	22.7	24.3	18.4	17.8

**Table 6:** Constitutive repeating unit (CRU) for various oxidation products formed in PE. Corresponding values for the molar mass ($M_{\mathrm{CRU}})$ and the total number of atoms of the CRU ($N_{\mathrm{CRU}}$ ), the number of O_2_ molecules chemically consumed per carbon atom ($n_{O2}$ ) and three key ratios: ${\Delta M}_{a}/{\Delta n}_{O2}$, ${\Delta M}_{a}/{\Delta Q}_{O2}$ and ${\Delta \rho }_{a}/{\Delta Q}_{O2}$.

CRU	$M_{\mathrm{CRU}}\;(g\cdot {\mathrm{mol}}^{-1})$	$N_{\mathrm{CRU}}$	$n_{O2}$	$\Delta M_{a}/\Delta n_{O2}\;(g\cdot {\mathrm{mol}}^{-1})$	$\Delta M_{a}/\Delta Q_{O2}\;(g\cdot {\mathrm{cm}}^{3}\cdot {\mathrm{mol}}^{-2})$	$\Delta \rho_{a}/\Delta Q_{O2}\;(g\cdot {\mathrm{mol}}^{-1})$
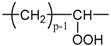	14p + 32	3p + 2	1/p	7.56	124.44	37.33
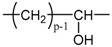	14p + 16	3p + 1	1/2p	7.56	124.44	37.33
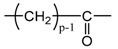	14p + 14	3p − 1	1/2p	12.44	234.24	70.27
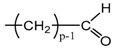	14p + 15	3p	1/2p	10.00	175.69	52.71
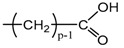	14p + 31	3p + 1	1/p	8.78	149.23	44.77
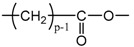	14p + 30	3p	1/p	10.00	175.69	52.71

**Table 7:** Comparison between the lifetimes determined using a dielectric or a mechanical end-of-life criterion for Si-XLPE in the various radio-thermal environments under study.

**I (Gy·h^−1^)**	400	77.8	8.5	6.0
**T (°C)**	21	47	47	86
$t_{F}\left(\varepsilon^{\prime }\right)$ **(days)**	67	184	629	289
$t_{F}\left(\varepsilon_{R}\right)$ **(days)**	32	43	246	-
** $\mathrm{Ratio}\;t_{F}\left(\varepsilon^{\prime }\right)/t_{F}\left(\varepsilon_{R}\right)\;$ **	2.1	4.3	2.6	-

**Figure A1:**
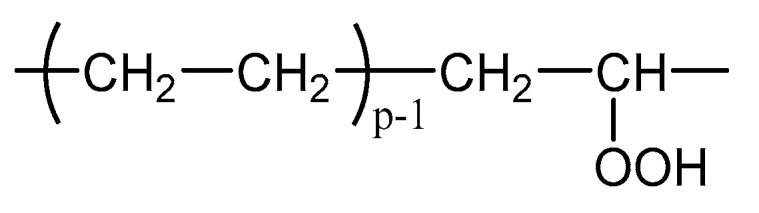
Constitutive repeating unit for hydroperoxidized PE.

## Data Availability

The data presented in this study are available on request from the corresponding author.

## Appendix A. Evaluation of the Effect of Oxygen Consumption on the PE Density When Hydroperoxides Are the Main Oxidation Products

The formation of hydroperoxides in PE can simply be described using the constitutive repeating unit (CRU) shown in Figure A1.

The molar mass and the total number of atoms of this CRU are:

(A1) $$M_{\mathrm{CRU}}=14p+32\;\mathrm{and}\;N_{\mathrm{CRU}}=3p+2$$

The total number of O_2_ molecules per carbon atom is:

(A2) $$n_{O2}=\frac{1}{p}$$

As a reminder, the average atomic molar $M_{a}$ is defined as:

(A3) $$M_{a}=\frac{M_{\mathrm{UCR}}}{N_{\mathrm{UCR}}}$$

Introducing Equations (A1) and (A2) into Equation (A3) gives:

(A4) $$M_{a}=\frac{14p+32}{3p+2}$$

i.e.,

(A5) $$M_{a}=\frac{7+16n_{O2}}{1.5+n_{O2}}$$

The derivative function of $M_{a}$ with respect to $n_{O2}$ is written as:

(A6) $$\frac{{\mathrm{dM}}_{a}}{{\mathrm{dn}}_{O2}}=\frac{17}{{\left(1.5+n_{O2}\right)}^{2}}$$

For the low conversion ratio of the oxidation reaction ($n_{O2}\ll 1$), this can finally be written as:

(A7) $$\frac{{\mathrm{dM}}_{a}}{{\mathrm{dn}}_{O2}}\approx 7.55$$

i.e., if considering small variations:

(A8) $$\frac{\Delta M_{a}}{\Delta n_{O2}}\approx 7.55\;g\cdot {\mathrm{mol}}^{-1}$$

In addition, oxygen consumption $Q_{O2}$ can be written as:

(A9) $$Q_{O2}=\frac{\rho_{a\;\mathrm{ini}}}{32}\frac{\Delta M_{\mathrm{UCR}}}{M_{\mathrm{UCR}\;\mathrm{ini}}}$$

where $M_{\mathrm{UCR}\;\mathrm{ini}}$ and $\rho_{a\;\mathrm{ini}}$ are the molar mass of the CRU ($M_{\mathrm{UCR}\;\mathrm{ini}}=14p$) and the density of the amorphous phase of the unoxidized PE ($\rho_{a\;\mathrm{ini}}=0.85$ [69]), respectively. Replacing $M_{\mathrm{UCR}}$ and $M_{\mathrm{UCR}\;\mathrm{ini}}$ with their respective expression gives:

(A10) $$Q_{O2}=\frac{\rho_{a\;\mathrm{ini}}}{14}n_{O2}$$

The derivative function of $Q_{O2}$ with respect to $n_{O2}$ is written as:

(A11) $$\frac{{\mathrm{dQ}}_{O2}}{{\mathrm{dn}}_{O2}}=\frac{\rho_{a\;\mathrm{ini}}}{14}$$

The numerical application gives:

(A12) $$\frac{{\mathrm{dn}}_{O2}}{{\mathrm{dQ}}_{O2}}\approx 16.47$$

i.e., if considering small variations:

(A13) $$\frac{\Delta n_{O2}}{\Delta Q_{O2}}\approx 16.47{\;\mathrm{cm}}^{3}\cdot {\mathrm{mol}}^{-1}$$

Combining Equations (A8) and (A13) gives:

(A14) $$\frac{\Delta M_{a}}{\Delta Q_{O2}}=\frac{\Delta M_{a}}{\Delta n_{O2}}\times \frac{\Delta n_{O2}}{\Delta Q_{O2}}=124.44\;g\cdot {\mathrm{cm}}^{3}\cdot {\mathrm{mol}}^{-2}$$

Let us choose the upper limit of the variation interval proposed by Langlois et al. [55]:

(A15) $$\frac{\Delta \rho_{a}}{\Delta M_{a}}\approx 0.30\;\mathrm{mol}\cdot {\mathrm{cm}}^{-3}$$

Combining Equations (A14) and (A15) finally gives:

(A16) $$\frac{\Delta \rho_{a}}{\Delta Q_{O2}}=\frac{\Delta \rho_{a}}{\Delta M_{a}}\times \frac{\Delta M_{a}}{\Delta Q_{O2}}=37.33\;g\cdot {\mathrm{mol}}^{-1}$$
